# Molecular Mechanism of Slow Vegetative Growth in *Populus* Tetraploid

**DOI:** 10.3390/genes11121417

**Published:** 2020-11-27

**Authors:** Congping Xu, Ying Zhang, Qiang Han, Xiangyang Kang

**Affiliations:** 1 Beijing Advanced Innovation Center for Tree Breeding by Molecular Design, National Engineering Laboratory for Tree Breeding, College of Biological Sciences and Technology, Beijing Forestry University, Beijing 100083, China; foreverxcp@126.com (C.X.); yingzhanga@bjfu.edu.cn (Y.Z.); hanqiang1988@163.com (Q.H.); 2 Key Laboratory of Genetics and Breeding in Forest Trees and Ornamental Plants, Ministry of Education, College of Biological Sciences and Technology, Beijing Forestry University, Beijing 100083, China; 3 Beijing Laboratory of Urban and Rural Ecological Environment, College of Biological Sciences and Technology, Beijing Forestry University, Beijing 100083, China

**Keywords:** *Populus*, tetraploid, miRNAs, dose effects, differential genes, endogenous hormones

## Abstract

Tetraploid plants often have altered rates of vegetative growth relative to their diploid progenitors. However, the molecular basis for altered growth rates remains a mystery. This study reports microRNA (miRNA) and gene expression differences in *Populus* tetraploids and counterpart diploids using RNA and miRNA sequencing. The results showed that there was no significant difference between young leaves in the expression of vegetative growth-related miRNAs. However, as leaves aged, the expression of auxin- and gibberellin-related miRNAs was significantly upregulated, while the expression of senescence-related miRNAs was significantly downregulated. The dose effect enhanced the negative regulation of the target genes with *ARFs*, *GA20ox*, *GA3ox*, and *GAMYB* being downregulated, and *TCP* and *NAC* being upregulated. As a result, the chloroplast degradation of tetraploid leaves was accelerated, the photosynthetic rate was decreased, and the synthesis and decomposition ability of carbohydrate was decreased.

## 1. Introduction

Whole genome duplication has been a major source of genetic diversity in the plant lineage [1,2,3]. In many cases, polyploidization results in altered growth, morphology, physiology, and biochemistry [2,4,5,6,7]. This genetic and phenotypic diversity makes the use of polyploidy in plant breeding an effective method for trait improvement and utilization [8].

An increasing number of studies have focused on polyploidy in plants. Some studies showed that tetraploids have a significant advantage in vegetative growth [6,9,10,11,12]. The plant height and the leaf area of mulberry (*Morus alba* L.) tetraploids were larger than those of diploids [9]. Tetraploids grew higher than diploids in *Medicago* and had larger somatic cells and biomass [10]. The growth rate and photosynthetic rate of tetraploids were higher than those of diploid *Phlox drummondii* [11]. Compared with diploids, the leaf area, chlorophyll content, and photosynthetic rate were larger in *Arabidopsis* tetraploids [12]. Ni et al. [6] found that the advantages of growth in *Arabidopsis* allotetraploids had a relationship with circadian clocks. During the daytime, the repressors *LHY* and *CCA1* were more inhibited in tetraploids than in their parents [6]. Then, the expression of genes related to chlorophyll synthesis, sucrose and starch synthesis, and metabolism were upregulated in *Arabidopsis* polyploids, which resulted in the accumulation of photosynthesis products and starch [6]. These findings suggest that tetraploids have more advantages than diploids in vegetative growth [6,9,10,11,12].

However, not all tetraploid plants have the same characteristics as *Arabidopsis thaliana* [13,14,15]. The leaf area and chlorophyll content of wheat tetraploids were higher than those of diploids, but the photosynthetic rate was lower than that of diploids [13]. The plant height of potato tetraploid plants was lower than that of diploids [14]. The height of tetraploid birch was shorter than that of diploid birch [15].

GA3-oxidase and GA20-oxidase are two key enzymes in the synthesis of gibberellin [16]. GA3-oxidase can suppress light-mediated hypocotyl elongation by inhibiting chlorophyll biosynthesis [17]. *GID1*, a gene for a gibberellin (GA) receptor, can improve the sensitivity to GA and involved in GA signaling [18]. IAA17, a repressor of auxin-inducible gene expression, positively modulates natural leaf senescence through melatonin-mediated pathway in *Arabidopsis* [19]. GH3 negatively regulates auxin synthesis and causes plant dwarfism [20]. *ARF6* and *ARF8*, two auxin response factors, regulate jasmonic acid biosynthesis and floral organ development [21,22]. Furthermore, expression of *ARFs* is controlled by miR167 [23]. *BRI1* is involved in brassinosteroid signal transduction, whereby it binds at an extracellular site and results in phosphorylation of the kinase domain, which activates the BRI1 protein leading to BR responses [24,25]. *PAO1*, *CLH1*, *CLH2*, and *POR* all play important roles in the process of chlorophyll synthesis [26]. *PSAF* and *PSBR* participate in photosynthesis and encode the subunits of PSⅠ (photosystemⅠ)and PS Ⅱ(photosystem Ⅱ), respectively [27,28]. *SUS*, *SS*, *AMY*, *EMB*, and *BAM* are all involved in starch synthesis and decomposition [29]. *SPS* promotes sucrose synthesis [30]. Gene expression analysis of dwarfed tetraploid apple plants found that *AUX1*, *ARF3*, *DWF4*, *BKI1*, and *BIN2* were significantly downregulated genes involved in the pathway of auxin and brassinolide [31].

Taken together, these studies suggest that vegetative growth rates between diploid and tetraploids is unpredictable, with some species showing slower or faster growth. An understanding of the molecular basis for these differences in growth rate may help in predicting the effects of ploidy in the future. Do similar molecular changes result in similar growth patterns in diploid and tetraploid varieties? The molecular mechanism needs to be further studied and clarified.

To answer this question, a number of *Populus* tetraploids were obtained by somatic chromosome doubling during in vitro culture leaf explants [32]. In this study, by selecting tetraploids and counterpart diploids as materials, microRNA (miRNA) and target gene expressions were studied in various canopy gradient leaves of *Populus* by RNA-seq and miRNA sequencing. Meanwhile, combined with the analysis of cytological, physiological, and biochemical indices, we clarified the molecular regulatory mechanism of tetraploids with slow vegetative growth in these tetraploids.

## 2. Materials and Methods

### 2.1. Plant Material

Eight genotypes of *Populus* tetraploids (2n = 4x = 76) (T-1, T-2, T-3, T-4, T-5, T-6, T-7, and T-8) and their counterpart diploids (2n = 2x = 38) (D-1, D-2, D-3, D-4, D-5, D-6, D-7, and D-8) were used in this study. The eight genotypes of tetraploid plants used in the study were previously induced by chromosome doubling of diploid hybrid progeny ((*Populus pseudo-simonii* × *P. nigra* Zheyin3#) × (*P.* × *beijingensis*)) through colchicine treatment [32]. Healthy shoots of tetraploid and counterpart diploid plants were grown for 30 days in half-strength MS (Murashige and Skoog) root medium (3% (*w*/*v*) sucrose, 0.6% (*w*/*v*) agar, 0.49 μM IBA (indolebutyric acid)) and then transplanted into substrate (sand, turf, and peat, 1:1:1 *v*/*v*/*v*) in plastic pots (25 cm in diameter × 25 cm in depth); we transplanted 15 uniform 2*x* and 4*x* seedlings of each genotype. All plant materials were grown in the greenhouse of the National Engineering Laboratory for Tree Breeding (Beijing, China) with 18/26 °C (night/day) and relative humidity of 45–70%.

### 2.2. Measurement of Plant Growth Rate, Leaf Greenness, Leaf Area, and Photosynthesis

Plant height (H) of 1 month old 2*x* and 4*x* plants was measured from the apex to the base using a ruler once a month. Total leaf greenness was measured in the 1st, 5th, 10th, 15th, and 20th leaves of 8 month old plants using a SPAD-502 Chlorophyll Meter Model (Minolta, Tokyo, Japan). Leaf area and Pn (net photosynthetic rate) were measured from the first fully expanded leaf on the stem (the diploids were marked as D_1, D_2, D_3, D_20, while the tetraploids were marked as T_1, T_2, T_3, T_20 from the top to the bottom of the stem). Leaf areas were measured using a portable area meter (LI-3100C, LI-COR, Lincoln, NE, USA). Pn was measured using the LI-6400-02B portable photosynthesis system (Li-COR-6400, Li-COR, Inc., Lincoln, NE, USA). Measurements were taken on sunny days at 8:30–11:30 a.m., with maintenance of the photosynthetic photon flux density at 1400 mol (photon)·m^−2^·s^−1^, a CO_2_ concentration of 400 mol·mol^−1^, relative air humidity of 60–65%, and flow of 500 mol·mol^−1^. Three biological replicates were used for each genotype.

### 2.3. Transcriptome Analysis

Measurement of biochemical characteristics on the 1st, 5th, and 15th leaves (as described above) showed that these characteristics changed significantly, implying that the substantial change in gene expression might relate to the leaf positions in *Populus* tetraploids. Therefore, the 1st, 5th, and 15th leaf samples for transcriptome sequencing in the tetraploid and diploid plants were harvested and placed into cryopreserved tubes, and then quickly frozen in liquid nitrogen. The TRIzol reagent kit (Invitrogen, Carlsbad, CA, USA) was used to extract the total RNA in leaf samples, and the RNase-free DNaseSet (Qiagen, Shanghai, China, https://www.qiagen.com/cn/) was used for the purification of RNA. Mixed gene pools were built with a mixture of eight candidate genotypes from the 1st, 5th, and 15th leaves of tetraploid and diploid plants; then, three independent biological replicates were sequenced and analyzed. RNAs of leaf samples were sequenced using Illumina paired-end technology and an Illumina HiSeq2000 platform (Life Technologies, Carlsbad, CA, USA) at the Beijing Yuanquanyike Biological Technology Co., Ltd. (Beijing, China). When the data were processed, original reads less than 60 bp in length were filtered first, and clean reads were mapped to the genome of *Populus trichocarpa* using TopHat (http://ccb.jhu.edu/software/tophat/index.shtml) [33] to generate read alignments for each sample. Genomic annotations were obtained from Phytozome (http://www.phytozome.net/). The transcript isoform level and gene level counts were calculated, and differential transcript expression was then computed using Cuffdiff (an algorithm that estimates expression at transcript-level resolution and controls for variability evident across replicate libraries) [33].

### 2.4. RT-PCR Validation of Differentially Expressed Genes (DEGs)

A total of 12 differential genes were analyzed for reverse transcription quantitative polymerase chain reaction (RT-qPCR). RT-qPCR was performed on a 7500 Fast Real-Time PCR System (AB Ltd., Waltham, Massachusetts, USA) using the SuperReal PreMix Plus (SYBR Green) kit (Tiangen Biotech CO., LTD, Beijing, China). The complementary DNA (cDNA) template for reactions was reverse-transcribed using total RNA extracted from leaves. The RT-PCR system included 2× SuperReal PreMix Plus, 10 L; forward primer, 0.6 L; reverse primer, 0.6 L; cDNA template, 2 L; 50× ROX Reference, 0.4 L; and Dye and RNase-free ddH2O, 6.4 L. RT-PCR was executed using the following process: 95 °C for 15 min for pre-degeneration, 95 °C for 10 s for degeneration, 58 °C for 30 s for annealing, and 72 °C for 30 s for extension. This process, excluding pre-degeneration, was then repeated 39 times, followed by 95 °C for 15 s and 60 °C for 1 min for dissolution curve analysis. All the samples and randomly selected genes were performed with four technical replicates and three biological replicates. The sequences of primers used in the present study were designed using an online tool called Primer3 Plus (http://www.primer3plus.com/) and are listed in Table S1 and Table S2 (Supplementary Materials). The constitutively expressed *Actin* (Accession number: EF145577) gene with stable expression was chosen as the reference gene, and the 2^−ΔΔCt^ method was used to calculate the gene expression by RT-PCR. All reactions were carried out in triplicate for biological repeats.

### 2.5. Gene Ontology (GO) Analysis

Gene Ontology (GO) is a standard gene function classification system based on molecular functions, biological processes, and cellular components. In this study, GO enrichment analysis of DEGs was implemented using the GO database (http://www.geneontology.org/) and the GO Term Finder (http://www.yeastgenome.org/help/analyze/go-term-finder). GO terms with a corrected *p*-value <0.05 were considered significantly enriched by DEGs.

### 2.6. Phytohormone Analysis

The contents of plant endogenous hormones including gibberellic acid (GA3), auxin (IAA), zeatin (ZT), abscisic acid (ABA), salicylic acid (SA), and jasmonate (JA) were determined in the 1st, 5th, 10th, 15th, and 20th leaves in tetraploids and diploids using the high-performance liquid chromatography/mass spectrometry (LC–MS) method described in Pan et al. [34]. Leaves were harvested and immediately frozen in liquid nitrogen and then transferred to a −80 °C refrigerator. The leaf tissue of each sample was ground to powder in liquid nitrogen, and 50 mg was transferred to a 2 mL centrifuge tube, followed by the addition of 500 µL of extraction agent (2-isopropyl alcohol, water, and hydrochloric acid = 2:1:0.002); the internal standard solution of plant hormones contained 0.25 ng/µL ZT, 8 µL; 0.2 ng/nL GA3, 25 µL; 0.5 ng/µL IAA, 10 µL; 1 ng/µL ABA, 50 µL; 0.2 ng/µL SA, 50 µL; and 0.2 ng/µL JA, 50 µL. The centrifuge tubes were then placed on the rolling bed at 4 °C, 100× g for 30 min before 1 mL of methylene chloride was added to each tube. The tubes were shaken for an additional 30 min, and the mixed solution was centrifuged at 4 °C, 12,000× *g* for 10 min. The lower supernatant was transferred to a clean centrifuge tube and dried with nitrogen gas. Then, 200 µL of methanol was added to dissolve the leaf sample. A 10 µL sample was used for LC–MS analysis using an AB Sciex QTRAP 5500 LC System (AB Sciex Pte., Waltham, MA, USA), with acetonitrile and 0.4% triethylamine solution (pH 3.5) used as the mobile phase. We had three biological replicates for each sample, and each replicate was performed with three technical replicates.

### 2.7. Measurements of Chlorophyll, Starch, and Sugar Contents

The contents of chlorophyll, starch, and sugars were measured in the 1st, 5th, and 15th leaves of the eight genotypes of tetraploid and diploid plants, with three biological replicates of each genotype. Chlorophyll extraction and analyses were performed as described by Ni et al. [6], except that 1 g of leaf tissue was used per biological replication. Chlorophyll content was calculated using the following formula: chlorophyll content (mg·g^−1^) = 8.02 × A_663_ + 20.20 × A_645_ [6].

The starch and sugar contents were also measured in the 1st, 5th, and 15th leaves of tetraploids and diploids. Ten percent of the crude extract was prepared using 0.1 g of powdered fresh leaf tissues, which were homogenized in 0.9 mL of 1× phosphate buffer (pH 7.2). The contents of starch, sucrose, fructose, and glucose were determined using the EnzyChromTM Assay Kits (BioAssay Systems Company, Hayward, CA, USA, https://www.bioassaysys.com/) and expressed as mg substance per gram fresh leaf.

### 2.8. Enzyme Assay

Similar to the determination of starch and sugar contents, 10% of the enzymatic crude extract was prepared first in the 1st, 5th, 10th, 15th, and 20th leaves of tetraploids and diploids. The activities of sucrose synthase (SUS), sucrose phosphate synthase (SPS), and amylase (AMS) were assayed according to the protocol of assay kits (Nanjing Jiancheng Bioengineering Institute, Jiangsu Province, China http://www.comin.biz/index.html). The activity of chlorophyllase was measured with a plant chlorophyllase kit (GeneTex, Inc., Irvine, CA, USA, http://www.genetex.com/). Total protein concentration was measured by Bradford method, using bovine serum albumin as the standard. POD (peroxidase)activity was measured using a plant POD assay kit (Nanjing Jiancheng Bioengineering Institute). Malondialdehyde content was determined according to the method of Tripathi D.K. et al. [35].

### 2.9. Analysis of Mesophyll Cell Suspensions to Count Chloroplasts

The 1st, 5th, 10th, 15th, and 20th leaves were cut into 0.5–1 mm^2^ pieces and transferred to 10 mL of modified enzyme solution (0.6 M mannitol, Cellulase Onozuka R-10 (3% *w*/*v*), Macerozy ME R-10 (0.2% *w*/*v*), Pectolyase Y-23 (0.05% *w*/*v*)) at 25 °C for 3 h [36]. The number of chloroplasts was counted under a 40× lens using ultraviolet (UV) or bright-field illumination (Olympus BX51 microscope, Tokyo, Japan).

### 2.10. Transmission Electron Microscopy (TEM) Observations of Chloroplasts

The ultrastructure of chloroplasts in the 1st, 5th, 10th, 15th, and 20th leaves was prepared using the method of Otegui et al. [37] with minor modifications. The chloroplast ultrastructure was observed by JEM-1010 electron microscopy (JEM-1010, Jeol Ltd., Tokyo, Japan).

### 2.11. Statistical Analysis

Statistical analyses of physiological data were performed using SPSS ver. 19.0. software (SPSS Inc., Chicago, IL, USA). Significance of differences among means was determined by Duncan’s multiple-range tests at *p* ≤ 0.05.

## 3. Results

### 3.1. Comparative Analysis of Plant Height, Leaf Area, and Photosynthetic Rate between Tetraploid and Diploid Plants

**Figure 1:**
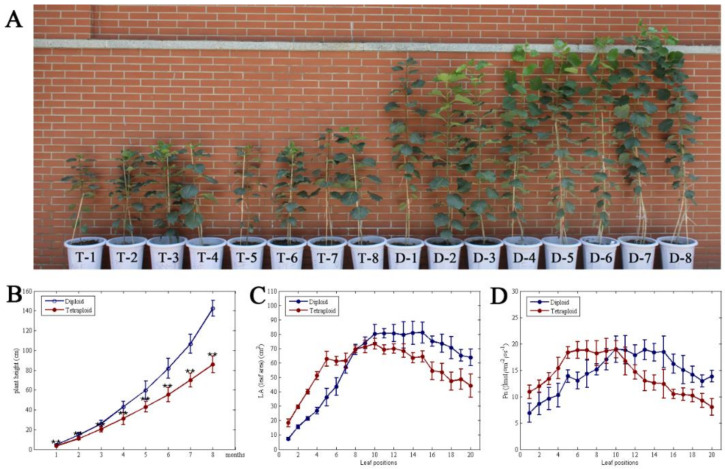
Comparison of plant height and Pn (net photosynthetic rate) of *Populus* tetraploids and diploids. (**A**) Phenotype of *Populus* tetraploid and diploid after 6 months. (**B**) Average growth rates (height) between tetraploids and diploids during time. (**C**) Leaf area at different leaf canopy positions in tetraploid and diploid plants. (**D**) Photosynthesis in tetraploid and diploid plants at different leaf positions.

After 8 months of growth, the plant height of *Populus* diploids was 33.1%, 33.7%, 26.0%, 37.1%, 38.6%, 48.0%, 52.3%, and 66.2% higher than that of tetraploids for every month (Figure 1B). This indicates that the growth rate of *Populus* diploids is higher than that of tetraploids, and the gap in plant height is widened with increasing growth time (Figure 1A,B). For the top 10 leaves, the leaf area and photosynthetic rate in tetraploids were 150.98‒0.17% and 57.53‒9.50% higher than in the corresponding leaves of diploids, respectively (Figure 1C,D; Table S3, Supplementary Materials). However, with the increase in leaf age, the leaf area and net photosynthesis of tetraploids were decreased by 16.07–49.32% and 12.75–48.66%, respectively (Figure 1C,D). In addition, the total leaf number of tetraploids was 75% less than that of diploids, and the total photosynthetic rate was 34.48% lower than in diploids (Table S3, Supplementary Materials).

### 3.2. Verification of DEG Analysis Results by RT-PCR

**Figure 2:**
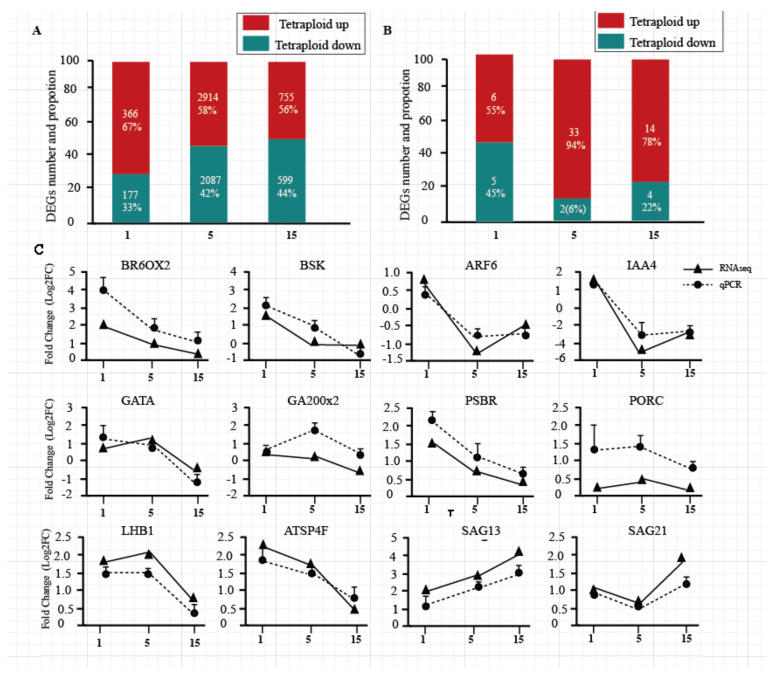
Differentially expressed genes (DEGs) and RT-PCR validation. (**A**) The numbers and proportions of upregulated and downregulated DEGs in the 1st, 5th, and 15th leaves of tetraploid plants. (**B**) The numbers and proportions of upregulated and downregulated microRNAs (miRNAs) in the 1st, 5th, and 15th leaves of tetraploid plants. (**C**) Confirmation of expression profiles of these genes using qRT-PCR. The fold changes in expression values for qRT-PCR were calculated by comparing the expression values of genes in 1st, 5th, and 15th leaves of tetraploid and diploid plants using the 2^−ΔΔCt^ method. The absolute fold changes were converted to log_2_ fold change (FC). Data are presented as means ± standard deviation (SD) from three independent experiments.

Compared with diploids, *Populus* tetraploids have 6898 differentially expressed genes (DEGs) in total, while 543, 5001, and 1354 were detected in the 1st, 5th, and 15th leaves, respectively. The number of upregulated genes was more than the number of downregulated genes in the three leaf positions of tetraploids (Figure 2A). DEGs and DEmiRNAs (differently expressed miRNAs) on different leaf canopy gradients were randomly selected to be verified by RT-PCR; the results showed the same tendency in most DEGs and DEmiRNAs, compared to RNA-seq data (Figure 2C; Figure S1, Supplementary Materials).

### 3.3. DEGs Associated with Hormone Synthesis and Signal Transduction in Tetraploids and Diploids

**Figure 3:**
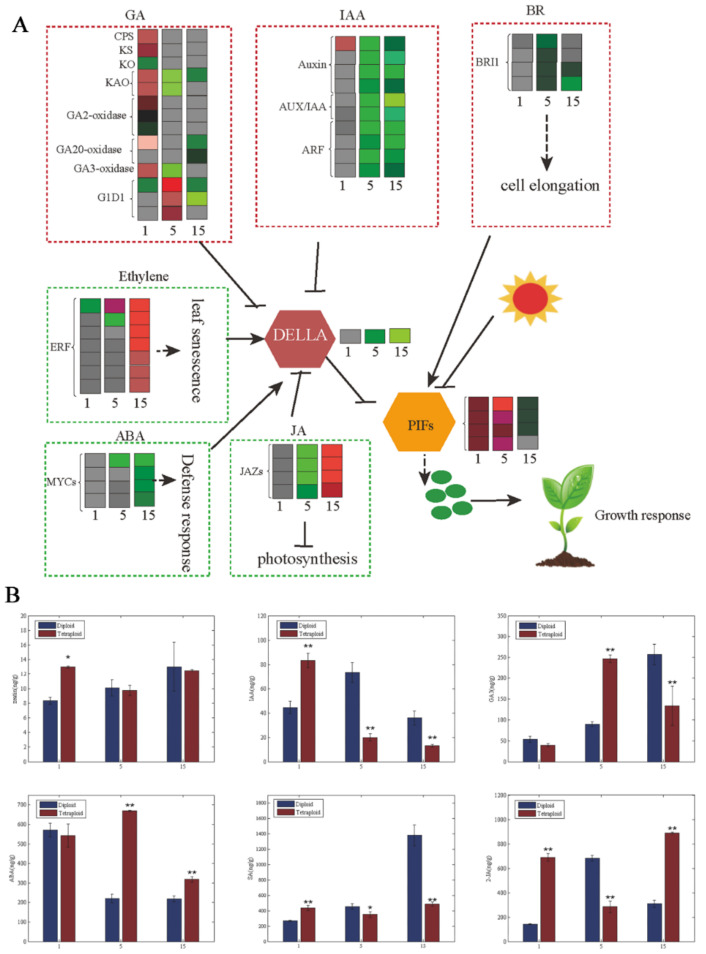
DEGs of hormonal pathways at the transcript level causing changes to hormones in tetraploids. (**A**) Overview of hormonal pathways and their crosstalk occurring at the transcript level in tetraploids compared to diploids. Red- and green-colored squares represent up- and downregulated genes (log2|FC| (≤−0.5 and ≥0.5)), respectively. The color saturation indicates log_2_ fold change between −2 and 2. (**B**) zeatin (ZT), auxin (IAA), gibberellic acid (GA3), abscisic acid (ABA), salicylic acid (SA), and jasmonic acid (JA) content at different leaf canopy positions of tetraploid and diploid plants; * *p* < 0.05, ** *p* < 0.01.

We found hormone synthesis and signal transduction to be important in tetraploid plant growth according to GO enrichment (Figure S2, Supplementary Materials). A total of 39 genes related to hormone synthesis and signal transduction showed differential expression between tetraploids and diploids (Figure 3A). Among the DEGs related to gibberellin synthesis and signal transduction, most genes, such as *CPS*, *KS*, *KAO*, and *GA3-oxidase*, were upregulated in the first leaves of tetraploids. However, most of the DEGs were downregulated in the fifth and 15th leaves of tetraploids, except for several genes, such as *KAO*, *GA3-oxidase*, *GA2-oxidase*, and *GID1*. The DEGs associated with auxin signal transduction were downregulated in the 1st, 5th, and 15th leaves, including auxin, Aux/IAA, and *ARFs*, except for the upregulated expression of auxin in the first leaves of tetraploids. The DEGs related to BR synthesis and signal transduction were downregulated in the fifth and 15th leaves of tetraploids, such as the BRI1 gene; there were no DEGs in the first leaves of tetraploids. In DEGs related to ethylene signal transduction, *ERF* expression was significantly downregulated in the first and fifth leaves but significantly upregulated in the 15th leaves of tetraploids. The expression of *JAZs* in the JA signaling pathway was similar to *ERF*. There were no DEGs related to ABA signal transduction in the first leaves of tetraploids. Most of the genes related to ABA signal transduction were upregulated in the fifth and 15th leaves of tetraploids, except for the *MYC* genes. The hormone results showed that the content of Zeatin, IAA, GA3, SA, and JA decreased gradually with an increase in leaf age, but the content of ABA increased gradually (Figure 3B). This finding was consistent with the expression of related DEGs.

### 3.4. DEGs Related to Chlorophyll Synthesis and Decomposition between Tetraploids and Diploids

**Figure 4:**
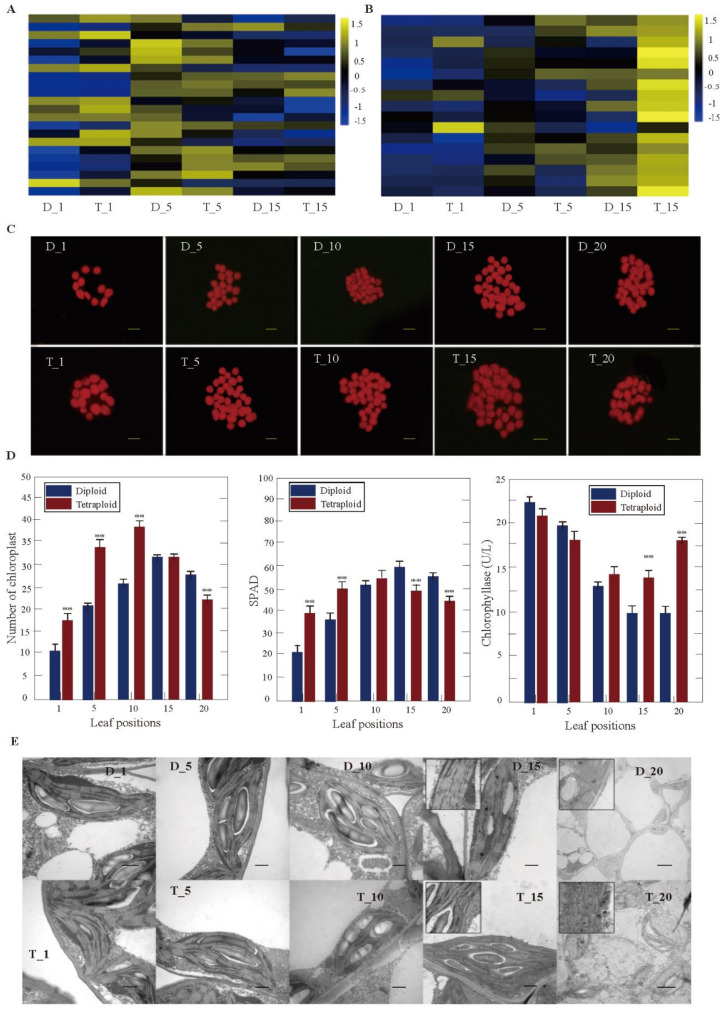
Chlorophyll synthesis and degradation in tetraploid and diploid plants. (**A**) Differentially expressed genes (DEGs) involved in chlorophyll synthesis at different leaf canopy positions when tetraploid plants were compared to diploid plants. (**B**) Differentially expressed genes (DEGs) involved in chlorophyll degradation at different leaf canopy positions when tetraploid plants were compared to diploid plants. (**C**) Comparison of the number of chloroplasts in mesophyll cells in different leaf positions of tetraploid and diploid plants. (**D**) The number of chloroplasts, SPAD (Soil and Plant Analyzer Development), and chlorophyllase activity in tetraploid and diploid plants; ** *p* < 0.01. (**E**) Chloroplast ultrastructure in different leaf positions of tetraploid and diploid plants.

GO enrichment found that chlorophyll synthesis and degradation play an important role in the growth difference between diploid and tetraploid plants (Figure S2, Supplementary Materials). There were 22 and 17 genes related to chlorophyll synthesis and decomposition in *Populus* tetraploids, respectively (Figure 4A,B). In DEGs related to chlorophyll synthesis, the expression of *LHCB3*, *LHB1B1*, *ELIP*, and *PORC* was significantly higher in the first leaves of tetraploids than in diploids. The number of genes significantly upregulated was almost equal to the number of downregulated genes in the fifth leaves of tetraploids (two genes upregulated, three genes downregulated). The expression of *PIFI* and *ELIP* was significantly higher in the 15th leaves of tetraploids than in diploids (Figure 4A). In DEGs related to chlorophyll decomposition, a few genes were differentially expressed in the first and fifth leaves of tetraploids, such as *PAO1*, *CLH1*, and *CLH2*. However, the expression of nine genes, such as *PAOs* and *CLHs*, was significantly higher in the 15th leaves of tetraploids than in the corresponding leaf position of diploids (Figure 4B).

The number of chloroplasts in the mesophyll cells of the 1st, 5th, and 10th leaves of tetraploids was significantly increased compared to diploids, and there was no significant difference in the 15th leaf position. However, the number of chloroplasts in mesophyll cells of the 20th leaves of tetraploids was significantly decreased compared to diploids (Figure 4C). At the same time, the chlorophyllase activity on the 15th and 25th leaves in tetraploids was 29.00% and 40.80% higher than in diploids, respectively (Figure 4D). There was no difference between tetraploid and diploid for the chloroplast ultrastructure of the 1st, 5th, and 10th leaves. However, there was a wider range of osmiophilic granules, and the stroma lamella was seriously damaged in the 15th leaves of tetraploids, which was not found in the leaves of diploids at the same position. This phenomenon was more obvious in the 20th leaves of tetraploids. Most chloroplasts in the cytoplasm were already degraded in the 20th leaves of tetraploids, but the stroma lamella was only slightly disordered in the 20th leaves of diploids, indicating that the chloroplast aging rate was faster in tetraploids than in diploids (Figure 4E).

### 3.5. DEGs Related to Photosynthesis between Tetraploids and Diploids

**Figure 5:**
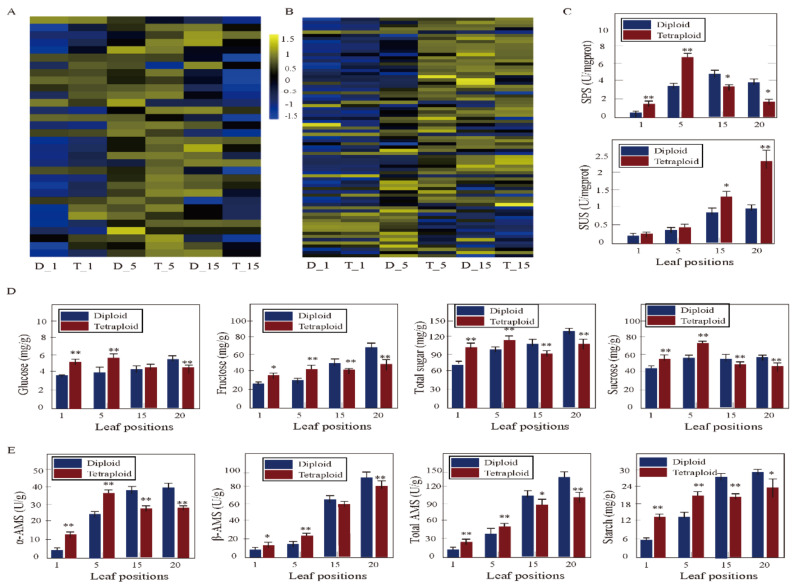
Photosynthesis, starch and sucrose metabolism in tetraploid and diploid plants. (**A**) Differentially expressed genes (DEGs) involved in light reaction and carbon fixation at different leaf canopy positions when tetraploid plants were compared to diploid plants. (**B**) Differentially expressed genes (DEGs) associated with sucrose and starch synthesis and metabolism at different leaf canopy positions when tetraploid plants were compared to diploid plants. (**C**) Sucrose synthase (SUS) and sucrose phosphate synthase (SPS) enzyme activity at different leaf canopy positions when tetraploid plants were compared to diploid plants. (**D**) Glucose, fructose, total sugar and sucrose content at different leaf canopy positions when tetraploid plants were compared to diploid plants. (**E**) Amylase (AMS) enzyme activity and starch content at different leaf canopy positions when tetraploid plants were compared to diploid plants; * *p* < 0.05, ** *p* < 0.01.

Photosynthesis was also momentous in tetraploid *Populus* growth (Figure S2, Supplementary Materials). There were 32 genes related to photosynthesis that showed differential expression (Figure 5A) between *Populus* tetraploids and diploids. Eight and seven genes were significantly upregulated in the first and fifth leaves of tetraploids compared with diploids, respectively, including *LHC* family genes involved in light trapping and *Psa* and *Psb* genes. The number of upregulated genes was significantly higher than that of downregulated genes. However, 19 genes were downregulated in the 15th leaves of tetraploids, including *PSBR*, *PIF4*, *PSAF*, *ENP*, *NPH3*, *NPL1*, and *PETC*. The results are consistent with the sequencing results. The photosynthetic rate of the first and fifth leaves of tetraploids was higher than that of diploids, but the photosynthesis of leaves was lower than that of diploids after the 10th leaf (Figure 1D).

### 3.6. DEGs Associated with Sucrose and Starch Metabolism between Tetraploids and Diploids

Sucrose and starch metabolism were also critical in tetraploid growth (Figure S2, Supplementary Materials). A total of 75 genes associated with sucrose and starch metabolism showed differential expression at different leaf canopy positions of *Populus* tetraploids compared with diploids. With increasing leaf age, the upregulated DEGs increased first and then decreased. Twenty-four genes in the fifth leaves of tetraploids were differentially expressed, including genes involved in starch synthesis and decomposition, such as *SUS*, *SS*, *AMY*, *EMB*, and *BAM*. However, the number of upregulated DEGs in the 15th leaves of tetraploids was significantly decreased, with only five genes, such as *UGT85A3* and *SUS5* (Figure 5B). At the same time, sugar and starch content, and related enzyme activity were detected. The results showed that starch, sucrose, glucose, and fructose contents, as well as SUS, SPS, α-AMS, and β-AMS activity, were significantly higher in the first and fifth leaves of tetraploids compared to diploids in *Populus*. However, the above indices in the 15th and 20th leaves of tetraploids were lower than in diploids. For diploids, the starch, sucrose, glucose, and fructose contents increased with increasing leaf canopy gradients. However, these indicators began to decline from the 15th leaves in tetraploids (Figure 5D–F).

### 3.7. Expression of Circadian Clock Genes in Different Leaf Canopy Positions

**Figure 6:**
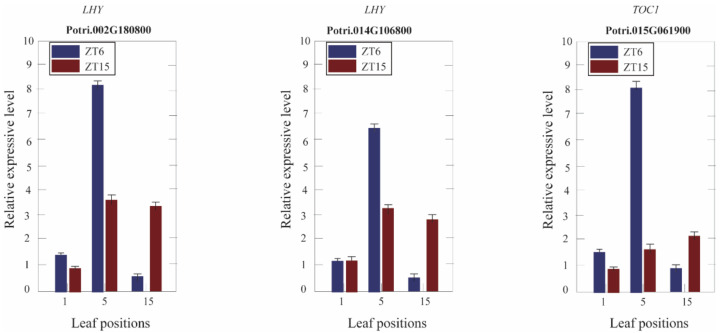
Quantitative RT-PCR analysis of the circadian clock-related genes at various leaf positions of *Populus* tetraploids.

In this study, the expression of *LHY* and *GI*, which were upstream of the transcription factor, showed no differences in the 1st, 5th, and 15th leaves between *Populus* tetraploid and diploid leaves at ZT4 (9:00 a.m.). The expression of *TOC1*, which was the downstream gene, was significantly upregulated in the fifth leaves of tetraploids, but there was no significant difference in first and 15th leaves of tetraploids (Table S4, Supplementary Materials). To investigate circadian clock gene regulation changes in *Populus* tetraploids, the expression of *LHY* and *TOC1* was determined by RT-PCR at ZT6 (12:00 a.m.) and ZT15 (9:00 p.m.), as shown in Figure 6. At ZT6 (12:00 a.m.) and ZT15 (9:00 p.m.), the expression of *LHY* showed no differences in the first leaves and was significantly upregulated in the fifth leaves of tetraploids. There was no significant difference in the 15th leaves at ZT6, but *TOC1* was significantly upregulated in the 15th leaves at ZT15. The expression of *TOC1* was significantly upregulated in the first leaves at ZT6, significantly upregulated in the fifth leaves of tetraploids at ZT6 and ZT15, and significantly upregulated in the 15th leaves at ZT15.

### 3.8. Differential Expression of miRNA between Tetraploids and Diploids

**Figure 7:**
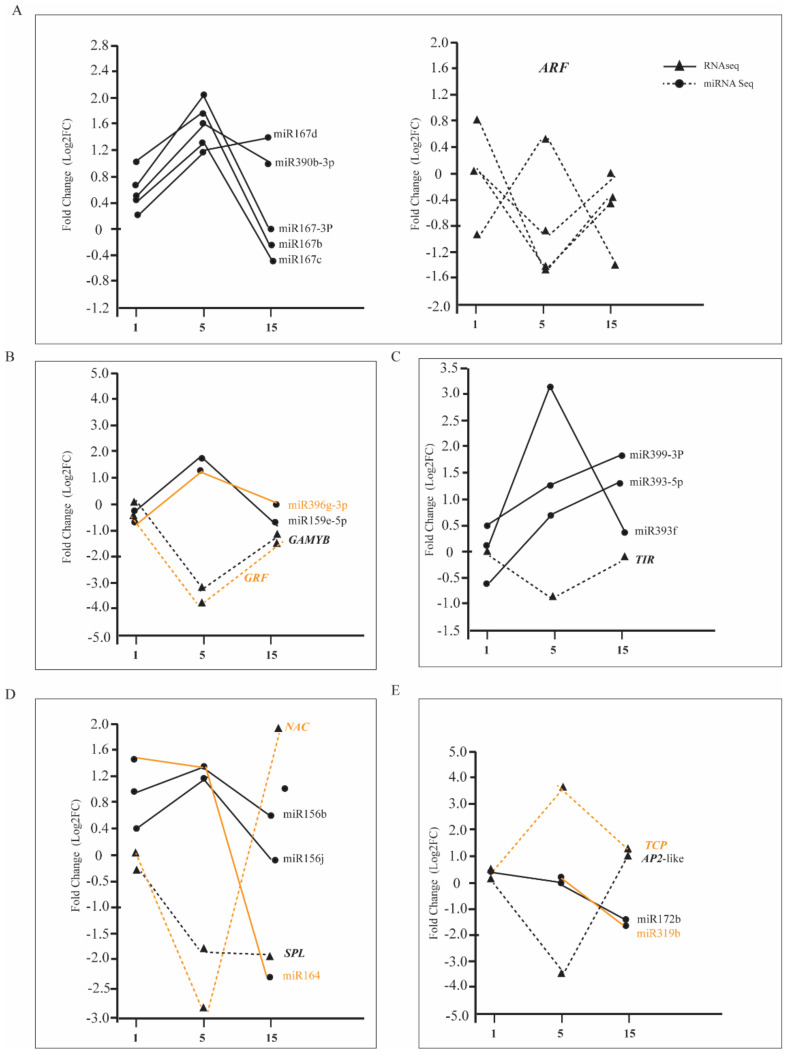
Compared with diploid, the expression of miRNAs and their target genes in different leaf positions of tetraploids. Solid and dashed lines of the same color are miRNAs and corresponding target genes. (**A**) The expression of miRNA167s and miRNA390 and their target genes *ARF*. (**B**) The expression of miRNA156s and miRNA164 and their corresponding target genes *GAMYB* and *GRF*. (**C**) The expression of miRNA393 and miRNA399 and their target genes *TIR*. (**D**) The expression of miRNA156s and miRNA164 and their corresponding target genes *SPL*and *NAC*. (**E**) The expression of miRNA172 and miRNA319 and their corresponding target genes *AP2-like* and *TCP*.

A total of 11, 35, and 18 differentially expressed of miRNAs were detected in the 1st, 5th, and 15th leaves of tetraploids, respectively (Table S5, Supplementary Materials). Among these miRNAs, the number of upregulated differentially expressed of miRNAs was higher than that of downregulated miRNAs in the fifth and 15th leaves of tetraploids (pcrB). With the increase in leaf age, the expression of miR167, miR390, miR393, miR399, miR159, and miR156, which are involved in the regulation of plant vegetative growth, was significantly higher than that of diploids (Figure 7). Therefore, the expression of the corresponding target genes *ARF*, *TIR*, *GAMAY*, *GRF*, and *SPL* was significantly reduced. In addition, the expression of miR530, miR164, miR172, and miR319, which are involved in the JA signaling pathway, vegetative growth stage transition, and leaf senescence, was significantly lower than in diploids. Therefore, the expression of the corresponding target genes *ERF*, *NAC*, *AP2*, and *TCP* was significantly increased (Figure 7).

## 4. Discussion

In this study, the growth rate of *Populus* tetraploids was significantly lower than that of diploids, which was similar to tetraploids of maize, potato, apple, willow, birch, and citrus [7,14,15,31,37,38]; however, the studies of tetraploids of *Arabidopsis thaliana*, *Phlox drummondii*, and mulberry (*Morus alba*) were not consistent [6,8,10]. Compared with diploids, *Populus* tetraploids exhibit slow growth. According to DEGs in different leaf canopy gradients between tetraploids and diploids, the most significant difference was that DEGs associated with vegetative growth increased first and then decreased with leaf growth development (Figure 4A and Figure 5A). However, DEGs negatively correlated with vegetative growth, which tended to increase gradually with increasing leaf age (Figure 4B and Figure 5B). The variation in DEGs should be an important reason for the slow growth of tetraploid *Populus*.

The growth and development of plants is regulated by endogenous hormones [28,29,30,31,39,40,41,42]. In our study, the effects of genome doubling on hormone synthesis and signal transduction were particularly prominent (Figure 3A). Compared with diploids, the GA3ox and GA20ox gene families that regulated gibberellin synthesis were downregulated gradually with increasing leaf age in *Populus* tetraploids (Figure 3A). At the same time, *IAA17*, *IAA4*, *ARF6*, *GH3*, and *IAA19* related to auxin synthesis, and *BRI1*, *BSK3*, and *BSK2*, related to brassinolide (BR), were downregulated with the increase in leaf age (Figure 3A). However, the genes related to ET, JA, and ABA biosynthesis, such as *ERF*, *JAZs*, *PLY*, and *PP2C* were upregulated with increasing leaf age. As a result, the contents of IAA, ZT, and GA3 decreased, while the contents of ET and ABA increased with increasing leaf age in tetraploid *Populus*. IAA, ZT, and GA3 promote cell division and stem elongation, and ET and ABA inhibit cell growth, which induces the senescence of tissues and organs [43,44,45,46,47,48]. These data indicate that the change in gene expression-related hormones in tetraploid *Populus* led to slow cell growth, thus accelerating leaf senescence.

Compared with diploids, protochlorophyllide oxidoreductase (POR) related to chlorophyll synthesis was downregulated in tetraploids. CHL, which is associated with chlorophyll degradation, was gradually upregulated in tetraploid *Populus* with increasing leaf age, which led to an increase in chlorophyll enzyme activity, a decrease in chlorophyll content, and accelerated chloroplast degradation; the degradation of chlorophyll and chloroplasts resulted in leaf senescence [49,50]. Corresponding to the changes in the genes related to chloroplast synthesis and degradation, two thylakoid membrane protein subunit compounds (Psa and Psb family members) and LHC family members that participated in light capture were upregulated in the first and fifth leaves of tetraploids, while the expression of these genes was downregulated in the 15th leaves of tetraploids. The results indicated that the leaf senescence rate and photosynthetic capacity of tetraploid *Populus* decreased faster than in diploids and affected the synthesis and decomposition of sugar and starch, which are the products of photosynthesis. The genes involved in the synthesis and metabolism of sucrose and starch, such as SUS, SPS, SS, AMS, BAM, and PHS, were mostly upregulated in the first and fifth leaves of tetraploids, but there was no significant difference in the 15th leaves between tetraploids and diploids. Apparently, changes in hormones in leaves led to regular changes in the expression of related genes, such as those involved in chlorophyll synthesis and degradation, photosynthetic carbon assimilation, and the synthesis and decomposition of sucrose and starch. Compared with diploids, the results showed the slow cell division and growth of tetraploids, the decrease in the number of leaves and chloroplast synthesis capacity, the acceleration in the degradation rate, and the decrease in sucrose and starch synthesis and decomposition, which eventually led to the tetraploid *Populus* being dwarfish.

What determines the variation of genes related to vegetative growth in tetraploids? Ni et al. [6] found a connection between the circadian clock and vegetative growth advantage in *Arabidopsis* allotetraploids. The circadian clock regulators and transcriptional repressors were inhibited more seriously during the day in *Arabidopsis* allotetraploids compared to diploids. From our results, the expression of *LHY* and *TOC1* showed differences in various leaf canopy gradients of *Populus* tetraploids, unlike *Arabidopsis* allotetraploids. For *CCA1*, there were no homologous genes in *Populus*, suggesting that the inhibition of *CCA1* might not work in tetraploid *Populus*. The results indicated that different circadian clock regulatory mechanisms might exist in different species.

Numerous studies have shown that noncoding RNAs, which are considered negative regulators, especially miRNAs, play an important role in plant growth and development, as well as cell differentiation [51,52]. Previous studies have found that miR167, miR390, miR393, and miR399 participate in auxin and BR signal transduction pathways by regulating transcription factor *ARFs* [31,53,54,55]. The target gene of miR159 is the transcription factor *GAMYB*, which regulates the synthesis of GA [43]. Tang et al. [56] reported that miR396 regulated plant height by influencing the synthesis and signal transduction of GA. miR164 and miR319 indirectly participate in the hormone synthesis pathways of JA and ET by regulating the transcription factors *NAC* or *TCP* and then regulating the process of leaf senescence. miR156 and miR172 participate in the growth and development of the leaves and regulate plant vegetative growth [50,51,52,53,54,55,56,57,58,59,60,61,62,63]. In this study, the expression of miR159, miR396, miR167, miR390, and miR399 increased with leaf age in tetraploid *Populus*, and the expression of *GAMYB*, *ARF*, *TIR*, and *GRF*, which are involved in the regulation of GA, auxin synthesis, and signal transduction, was significantly decreased (Figure 3A). However, the expression of miR530, miR164, miR172, and miR319 decreased with leaf age, and the expression of *ERF*, *NAC*, *AP2*, and *TCP*, which are involved in the regulation of JA and ET synthesis and signal transduction, was significantly upregulated (Figure 3A). The results showed that miRNA gradually showed a dose effect with increasing leaf age, which was closely related to the phenomenon of the leaf senescence of *Populus* tetraploids being faster than that of diploids.

## 5. Conclusions

**Figure 8:**
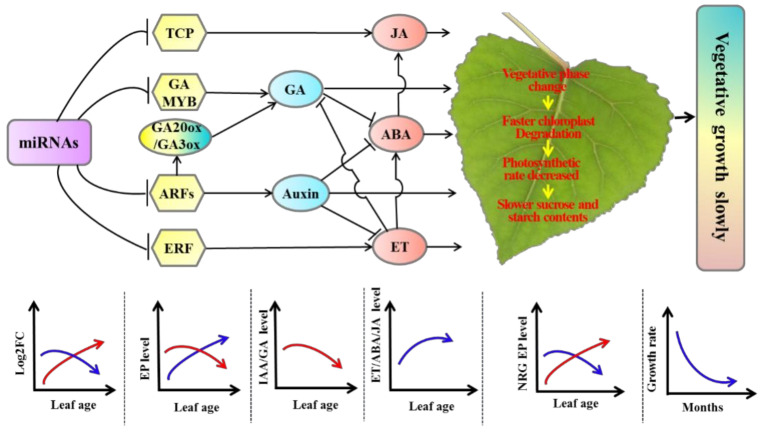
A proposed model illustrating the slow vegetative growth of *Populus* tetraploids. Model arrows and perpendicular lines indicate a promoting process and an inhibitory effect, respectively. The red curve represents the trend of miRNA expression of the target gene positively correlated with tetraploid vegetative growth or the trend of DEGs associated with vegetative growth. The blue curve represents the trend of miRNA expression of target genes negatively related to tetraploid vegetative growth or the trend of DEGs negatively related to vegetative growth.

The expression of miRNAs associated with the auxin and GA pathways increased with leaf age, which had a dosage effect in tetraploid *Populus*. Therefore, gene transcription and expression levels were inhibited by miRNAs, leading to relatively more negative control in target genes, such as *ARFs* and *GAMYB*, compared with diploid. In addition, the expression of miRNAs associated with the ET and JA pathways decreased with leaf age, leading to relatively less negative control in target genes by miRNAs, such as *ERF* and *TCP*, compared with diploid. The GA and IAA contents, which promote growth hormones, were significantly lower in tetraploids than diploids. However, the contents of JA and ET, which regulate senescence, were significantly higher in tetraploids than in diploids. As a result, leaf senescence in tetraploids was faster than in diploids, and the chloroplast aging rate was relatively faster than in diploids. The photosynthetic rate, as well as sucrose and starch synthesis metabolism, was decreased in tetraploids compared with diploids. Lastly, the *Populus* tetraploids grew more slowly than diploids (Figure 8).

## Supplementary Materials

The following are available online at https://www.mdpi.com/2073-4425/11/12/1417/s1: Table S1. The primer sequences for real-time PCR; Table S2. Primer sequences for miRNA real-time PCR; Table S3. Comparison of total net photosynthetic rate analysis of Populus tetraploid and diploid plants; Table S4. Different expression of circadian clock genes in the 1st, 5th, and 15th leaves of Populus tetraploids. Table S5. DifferentialLY expressed miRNA.
