# Supplementary material for: Molecular Mechanism of Slow Vegetative Growth in Populus Tetraploid
Source: Genes (Basel). 2020 Nov 27;11(12):1417. doi: 10.3390/genes11121417 (PMC7761321; doi:10.3390/genes11121417)
Supplement: Supplementary file 1 [file genes-11-01417-s001.zip › supplementary materials/Figure S1-S2.docx]

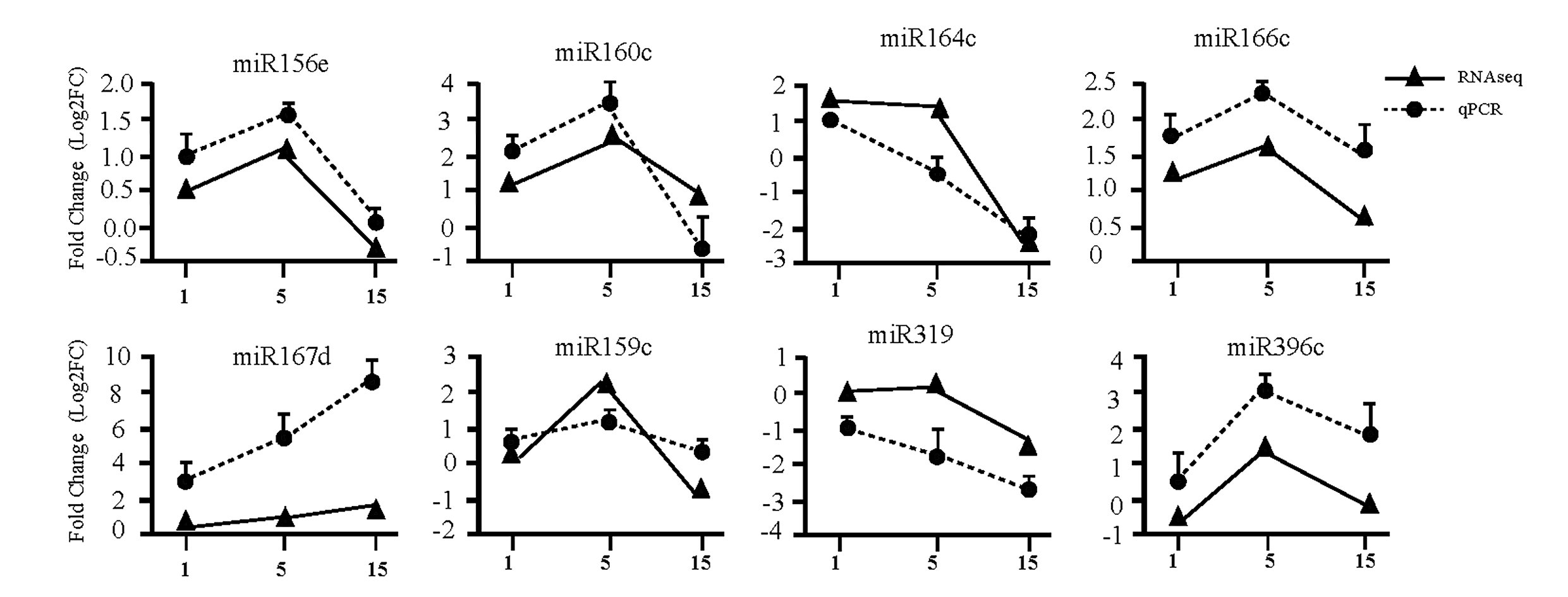


Figure S1. Confirmation of expression profiles of these miRNAs using qRT-PCR.The fold changes in expression values for qRT-PCR were calculated by comparing the expression values of miRNAs in 1st, 5th and 15th leaves of tetraploid and diploid plants using the 2−ΔΔCt method. The absolute fold changes were converted to Log_2_FC. Data are presented as means ± standard deviation (SD) from three independent experiments.


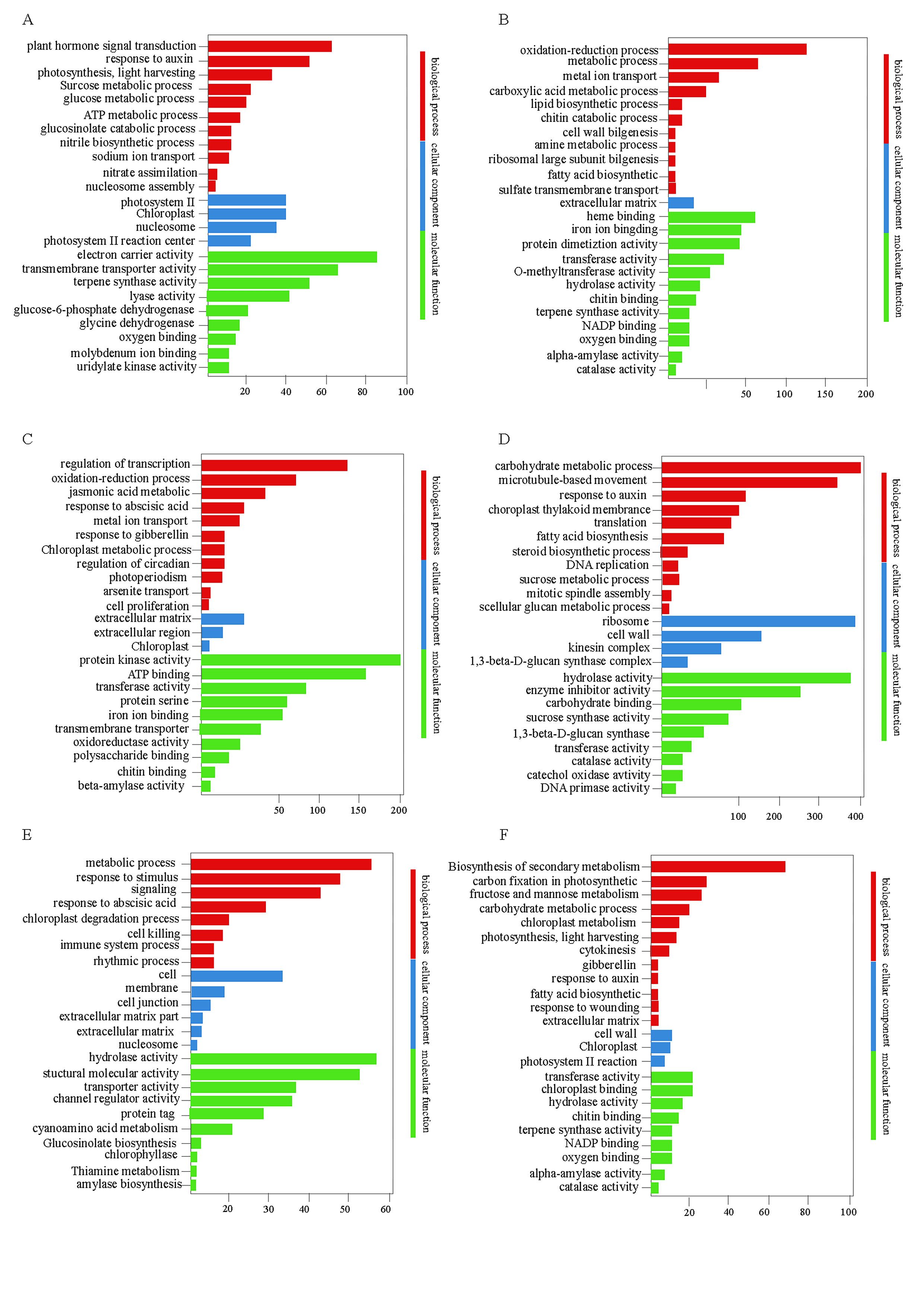


Figure S2. GO enrichment of differentially expressed genes (DEGs) in 1st, 5th and 15th leaves of tetraploid and diploid plants. (A) GO enrichment analysis of up-regulated DEGs in 1st of tetraploid. (B) GO enrichment analysis of down-regulated DEGs in 1st leaves of tetraploid. (C) GO enrichment analysis of up-regulated DEGs in 5th leaves of tetraploid. (D) GO enrichment analysis of down-regulated DEGs in 5th of tetraploid. (E) GO enrichment analysis of up-regulated DEGs in 5th leaves of tetraploid. (F) GO enrichment analysis of down-regulated DEGs in 5th of tetraploid.
