# Supplementary material for: Molecular Mechanism of Slow Vegetative Growth in Populus Tetraploid
Source: Genes (Basel). 2020 Nov 27;11(12):1417. doi: 10.3390/genes11121417 (PMC7761321; doi:10.3390/genes11121417)
Supplement: Supplementary file 1 [file genes-11-01417-s001.zip › supplementary materials/Tables S1-S4.docx]

Table S1. RT-PCR primer sequences

| **Gene ID** | **Forward primer** | **Reverse prime** |
| --- | --- | --- |

| *ACTINII-like* | | CTCCATCATGAAATGCGATG | CCACCTTGATTTTCATGCTGC | | |
| --- | --- | --- | --- | --- | --- |
| *LHY* | | TACAAAAGGTGTTCACTGGAGG | GGAATGATAGGCAAGCAAGCGAGGATAT | | |
| *LHY* | | AATCTGAAGGCTCATCTAACCGG | GAACGGAATGATAGACAAGGGAG | | |
| *TOC1* | GAAATCAGGCAGCAATGGAGGC | | | GAAGAGACAAATGGTGGAAC |  |
| *PSBR* | TCATCTTTCAAAGTTGAGGCTAGTCG | | | AACATAAACATCACCACTTGGAGACC |  |
| *LHB1* | CATTGGACATTTCAGTCAGGCAAC | | | CAAAAGAGGACCGGGGATCTATC |  |
| *POR C* | TTGGAGATTTGCGAGGTCTTGTGG | | | CTATGCAGCCAGGGTAGAGGGAAG |  |
| *SUS* | GCTGGGGTATATGGTTTTTGGAAG | | | CCGTGTGATAGTGCTGTGTCTGA |  |
| *ATSP4F* | ACCTTTCAGTTAGGTGGGGTATTG | | | GATGTTAGAGCTTTCTTGGGGGAC |  |
| *ARF6* | CTAGTGGGGGATGATCCTTGGGA | | | CCACGTGGAAGAAGAATTGTTGGC |  |
| *IAA4* | TGCAACAGAGCTTAGGTTAGGCC | | | GGTGGAGCAGTTTCTTGGTCATG |  |
| *BR6OX2* | TCCTGACCCATTCTCCTTTAACC | | | AGTCTCCTCCAACCTCTTCCCATC |  |
| *BSK* | AACTGGTTACAAAGATGAAGAGGG | | | GCAAAGACAGTTGCAGAAGGAATTG |  |
| *GATA* | CATCACCACAGCAAGCAGTGAGA | | | GAATTGGAATGCAGGGAAGGAACG |  |
| *GA20OX2* | CTCACTCTTGGCACTGGTCCTCAC | | | CTGTCCCTGTTCACCACTGCTCT |  |
| *SAG13* | TCTCTTCTGTTGTCGCATTTCTTTGC | | | TGCTCCGTACTATCTCTCTCTCAC |  |
| *SAG21* | CTTCCTCAAACGCAAAGGTCATCTC | | | CAGCAACATTCCCTGGTCTGTAG |  |

Table S2. Primer sequences of miRNA Realtime-PCR

| **MicroRNA name** | **Prime** |
| --- | --- |

| 5.8S | GTCTGCCTGGGTGTCACGCAA |
| --- | --- |
| miR396c | TCCACAGCTTTCTTGAACTC |
| miR159c | TTTGGATTGAAGGGATCTCC |
| miR156e | GCGCACAGAAGAGAGTGAGC |
| miR160c | GCTGCCTGGCTCCCTGTATG |
| miR164c | TGGAGAAGCAGGGCACGTTCA |
| miR167d | TGAAGCTGCCAGCATGATCTGAG |
| miR319 | CTTGGACTGAAGGGAGCTCCCTT |
| miR166c | TCGGACCAGGCTTCATTTTTA |

Table S3. Comparison total net photosynthetic rate analysis of *Populus* tetraploid and diploid

|  | **Diploid** | **Tetraploid** |
| --- | --- | --- |

| Total leaf area (cm^2^) | 1964.2±38.7^a^ | 1123.2±22.5 ^b^ |
| --- | --- | --- |
| Total leaf number | 35.0±0.89^a^ | 20.4±0.49^b^ |
| PE_w_ (µmol s^-1^) | 2.4±0.053^a^ | 1.6±0.022^b^ |

Note: Each value represents the mean ± SD of two experiments that involved at least 30 replicates. Values within the same column followed by different lower-case letters are significantly different based on a two-samplet t-test (*p*＜0.05).

Table S4. Different expression of circadian clock gene in the 1st、5th and 15th leaf of *Populus* tetraploid.

| **Leaf position** | **Gene symbol** | **Gene-ID** | **log_2_FC(T/D)** | **P value** | **Q value** |
| --- | --- | --- | --- | --- | --- |

|  | *LHY* | Potri.014G106800 | 0.037 | 0.6828 | 0.7446 |
| --- | --- | --- | --- | --- | --- |
|  |  | Potri.015G061900 | 0.17 | 0.0689 | 0.1052 |
| 1 | *TOC1* | Potri.005G196700 | 0.094 | 0.2821 | 0.3575 |
|  | *GI* | Potri.002G064400 | 0.085 | 0.3520 | 0.4317 |
|  |  | Potri.002G180800 | 0.064 | 0.4697 | 0.5495 |
|  | *LHY* | Potri.014G106800 | 0.42 | 5.00E-05 | 0.00012 |
|  |  | Potri.015G061900 | 0.21 | 0.0262 | 0.04429 |
| 5 | *TOC1* | Potri.005G196700 | 1.00 | 5.00E-05 | 0.00012 |
|  | *GI* | Potri.002G064400 | 0.50 | 5.00E-05 | 0.00012 |
|  |  | Potri.002G180800 | 0.42 | 5.00E-05 | 0.00012 |
|  | *LHY* | Potri.014G106800 | -0.089 | 0.3367 | 0.4157 |
|  |  | Potri.015G061900 | -0.018 | 0.8498 | 0.8836 |
| 15 | *TOC1* | Potri.005G196700 | 0.29 | 0.0012 | 0.0026 |
|  | *GI* | Potri.002G064400 | 0.37 | 5.00E-05 | 0.00012 |
|  |  | Potri.002G180800 | 0.12 | 0.1754 | 0.2387 |

Note: FC: fold change; T/D represented tetraploid/diploid; Bold font represented the differentially expressed genes in tetraploid which identified with the diploid in *Populus*.
